# Astrovirus MLB2, a New Gastroenteric Virus Associated with Meningitis and Disseminated Infection

**DOI:** 10.3201/eid2205.151807

**Published:** 2016-05

**Authors:** Samuel Cordey, Diem-Lan Vu, Manuel Schibler, Arnaud G. L’Huillier, Francisco Brito, Mylène Docquier, Klara M. Posfay-Barbe, Thomas J. Petty, Lara Turin, Evgeny M. Zdobnov, Laurent Kaiser

**Affiliations:** University of Geneva Hospitals, Geneva, Switzerland (S. Cordey, D.-L. Vu, M. Schibler, A.G. L’Huillier, K.M. Posfay-Barbe, L. Turin, L. Kaiser);; Geneva University Medical School, Geneva (S. Cordey, D.-L. Vu, M. Schibler, F. Brito, M. Docquier, K.M. Posfay-Barbe, T.J. Petty, L. Turin, E.M. Zdobnov, L. Kaiser);; Swiss Institute of Bioinformatics, Geneva (F. Brito, T.J. Petty, E.M. Zdobnov)

**Keywords:** high-throughput nucleotide sequencing, mamastrovirus, viral meningitis, viremia, immunocompromised host, viruses, astrovirus, astrovirus MLB2, enteric infections

## Abstract

Next-generation sequencing has identified novel astroviruses for which a pathogenic role is not clearly defined. We identified astrovirus MLB2 infection in an immunocompetent case-patient and an immunocompromised patient who experienced diverse clinical manifestations, notably, meningitis and disseminated infection. The initial case-patient was identified by next-generation sequencing, which revealed astrovirus MLB2 RNA in cerebrospinal fluid, plasma, urine, and anal swab specimens. We then used specific real-time reverse transcription PCR to screen 943 fecal and 424 cerebrospinal fluid samples from hospitalized patients and identified a second case of meningitis, with positive results for the agent in the patient’s feces and plasma. This screening revealed 5 additional positive fecal samples: 1 from an infant with acute diarrhea and 4 from children who had received transplants. Our findings demonstrate that astrovirus MLB2, which is highly prevalent in feces, can disseminate outside the digestive tract and is an unrecognized cause of central nervous system infection.

## Abstract 2

This virus is an unrecognized cause of central nervous system infection, particularly among immunocompromised patients.

Astroviruses, family *Astroviridae*, are small, nonenveloped, single-stranded RNA viruses. The family comprises 2 genera: *Mamastrovirus* species infect mammals, including humans, and *Avastrovirus* species infect poultry and other birds. Human astroviruses (HAstVs) were first identified in 1975 (*1*); until recently, only classic HAstVs that belonged to the species *Mamastrovirus* (MAstV) *1* were recognized as human pathogens. HAstVs contribute to ≈10% of nonbacterial, sporadic gastroenteritis in children, with the highest prevalence observed in community healthcare centers (*2*,*3*). Symptoms are generally mild, with patient hospitalization usually not required; asymptomatic carriage has been described in 2% of children (*4*).

Screening of fecal samples from persons with diarrhea and control samples in different parts of the world by unbiased next-generation sequencing (NGS) or reverse transcription PCR (RT-PCR) has revealed the sporadic presence of members of the *Astroviridae* family, previously unrecognized in humans, that are phylogenetically substantially distant from classic HAstVs (*3*,*5*–*9*). These viruses have been named HAstV-VA/HMO and HAstV-MLB, for Virginia, human-mink-ovine, and Melbourne, respectively, according to the place where they were first identified and their close phylogenetic distance to animal astroviruses; these viruses belong to distinct species (*10*). 

Cellular receptors and targeted cells for these viruses are unknown and, to date, novel astroviruses have not been culturable. Although the primary site of astroviral replication seems to be the gastrointestinal tract, disseminated diseases and encephalitis have been associated with infection with classic and nonclassic astroviruses (*11*–*16*). In animals, astroviruses also have the potential to target other organs; hepatitis and nephritis have been observed in avian infections (*4*,*17*).

These observations point to the noteworthy genetic diversity of astroviruses and their probable cross-species transmission. Nonetheless, clinical disease associated with new astrovirus variants remains to be confirmed (*9*,*18*). Although HAstV-MLB has been recovered from fecal samples of patients with acute flaccid paralysis (*6*), to our knowledge, no reports have documented this variant in cerebrospinal fluid (CSF) or central nervous system (CNS) tissue samples.

In June 2013, we launched a single-center prospective study using NGS to determine potential viral etiologic agents of meningoencephalitic and respiratory syndromes. Yet, in ≈50% of meningoencephalitis cases clinically suspected to be of viral origin, origins remain undetermined, despite comprehensive microbiologic investigations (*19*,*20*). We report the detection, in the context of this project, of an astrovirus MLB2 in the CSF of an immunocompetent adult patient with acute meningitis who was hospitalized at the University of Geneva Hospitals, Geneva, Switzerland, and the results of the pilot prevalence study and clinical investigation that this discovery triggered.

## Materials and Methods

### Virus Discovery Study

The virus discovery study and the pilot retrospective prevalence study it generated were approved by the University of Geneva Hospitals (CCER no. 13-075), and informed consent was obtained from the case-patient. This single-center epidemiologic study is ongoing (Technical Appendix 1).

### High-Throughput Sequencing and Sequence Analysis

High-throughput sequencing (RNA-seq library preparation, paired-end sequencing by using the 100-bp protocol with indexing on a HiSeq 2500 [Illumina, San Diego, CA, USA]) was performed directly on the case-patient’s CSF, plasma, urine, and anal swab specimen, and we analyzed results using the ezVIR pipeline as described (*21*). Of note, a DNA-seq library was also prepared and analyzed for the screening of CSF specimens of the virus discovery study.

We used high-throughput sequencing data from the anal swab specimen to obtain a MLB2 consensus sequence by aligning the reads from the ezVIR output with those of the MLB2 genome Bowtie2 (*22*) and then assembling them using Sparse Assembler (*23*). We used the full sequence (GenBank accession no. KT224358) and the capsid region (corresponding to nt 3831–6069 on the consensus sequence and nt 3843–6080 on the reference sequence) to perform a phylogenetic analysis. We made multiple alignments using multiple alignment with the fast Fourier transform (*24*) and built the tree using IQTree (*25*), with 10,000 bootstrap replicates. The tree was created with Evolview (*26*) using reference strains from GenBank (Technical Appendix 2 Tables 1, 2).

### Extraction and Construction of Specific Real-Time RT-PCR 

We spiked 190-μL patient specimens of CSF, plasma, urine, anal swab, and nasopharyngeal aspirates with 10 μL of standardized canine distemper virus of known concentration (*27*) and extracted RNA with the NucliSENS easyMAG (bioMérieux, Geneva, Switzerland) nucleic acid kit in an elution volume of 25 μL, according to the manufacturer’s instructions. We directly used extracted RNA for astrovirus MLB2–specific real-time RT-PCR screening analysis using an assay described by L.R. Holtz et al. (*11*). We performed PCR assay reaction using the QuantiTect Probe RT-PCR Kit (QIAGEN, Valencia, CA, USA) on a StepOnePlus instrument (Applied Biosystems, Rotkreuz, Switzerland) under the following cycling conditions: 50°C for 30 min, 95°C for 15 min, 45 cycles of 15 s at 94°C, and 1 min at 55°C. Data were analyzed with the StepOne software V.2 (Applied Biosystems). Analytical sensitivity was assessed with a plasmid including the target region and showed a limit of detection corresponding to 25 copies/reaction. Positive specimens were further analyzed for confirmation with a second real-time RT-PCR targeting the viral RNA-dependent RNA polymerase region (forward primer 5′-TCCCTTCTGGTGAGGTCACTCT-3′, reverse primer 5′-AGGCTTGCAACCAATAGTTAATCAT-3′, and probe 5′-FAM-AACCGTGGTAATCCATCCGGTCAAATATCA-TAMRA-3′) under the following cycling conditions: 50°C for 30 min, 95°C for 15 min, 45 cycles of 15 s at 94°C, and 1 min at 60°C.

### Pilot Prevalence Study

To estimate the local prevalence of this novel astrovirus, we tested some CSF specimens, including all of those with a total leukocyte count of >5 cells/μL, collected from April 2013 through April 2015, and all fecal specimens collected from August 2014 through August 2015 with the astrovirus MLB2–specific real-time RT-PCR targeting the capsid gene. Specimens were collected from pediatric and adult patients hospitalized at the University of Geneva Hospitals, a 1,900-bed tertiary-care medical center, and sent to the center’s laboratory of virology for any clinical purpose. All samples had been stored at −80°C.

## Results

### Astrovirus MLB2 in Case-Patient 

**Figure 1:**
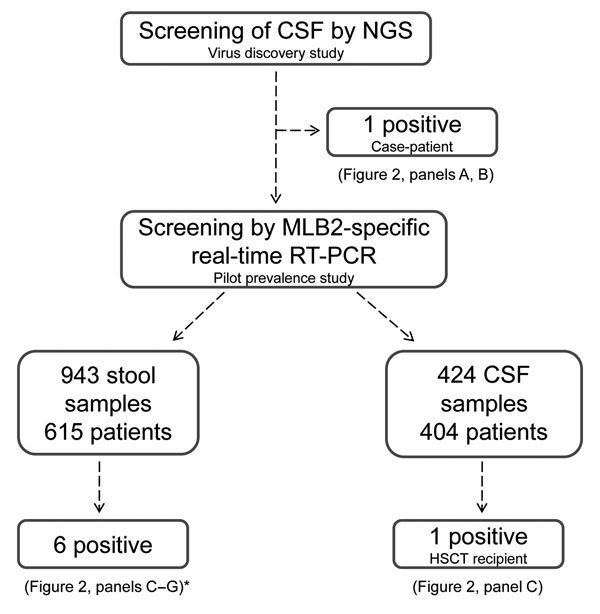
Flowchart of study using NGS to determine potential viral etiologic agents of meningoencephalitic and respiratory syndromes, Geneva, Switzerland, 2014. *The diarrheic immunocompetent infant is not represented in Figure 2. CSF, cerebrospinal fluid; NGS, next-generation sequencing; RT-PCR, reverse transcription PCR.

**Figure 2:**
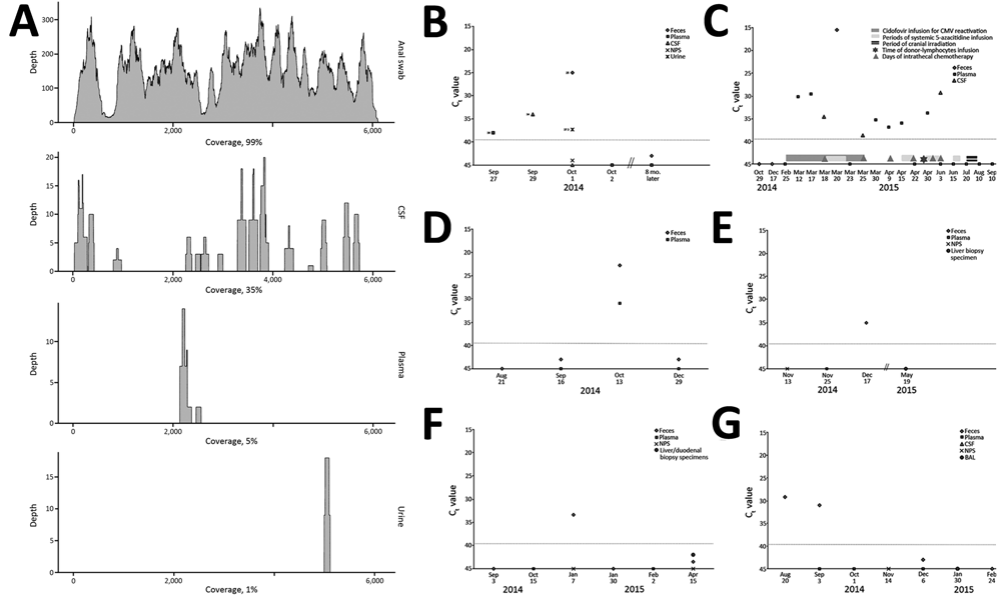
Details of the cases of astrovirus MLB2 infection, Geneva, Switzerland, 2014. A) Next-generation sequencing results for the case-patient. Read coverage histogram is shown for each specimen analyzed. Percentage of genome coverage is also indicated. B–C) Real-time RT-PCR analysis results for the case-patient (B) and the HSCT recipient (C); D–G) real-time RT-PCR analysis results for the solid organ transplant pediatric recipients: liver transplant (D–F) and kidney transplant (G). Panel A) Red dashed lines represent the limit of PCR positivity (cycle threshold 40). CSF, cerebrospinal fluid; NPS, nasopharyngeal swab; BAL, bronchoalveolar lavage.

The CSF collected from a patient with acute meningitis who was enrolled in the virus discovery study tested positive for astrovirus MLB2 (Figure 1) with a total of 155 specific reads (35% genome coverage; total covered, 2,183 bp). Reads did not map to other RNA viruses, and DNA sequencing revealed no reads for bacterial or viral pathogens. For this case-patient, astrovirus MLB2–specific reads were further detected by NGS in the following acute-phase specimens: anal swab (70,890 reads, 9,340 after duplicate removal; 99% genome coverage; total covered, 6,107 bp); plasma (18 reads, 5% genome coverage; total covered, 336 bp); and urine (16 reads; 1% genome coverage; total covered, 120 bp) (Figure 2, panel A).

CSF obtained at hospital admission was confirmed positive by astrovirus MLB2–specific real-time RT-PCR targeting the capsid gene (*11*). Anal swab and urine specimens collected during the acute phase were also confirmed positive by astrovirus MLB2–specific RT-PCR, with the highest viral load found in the anal swab specimen. The plasma specimen drawn at admission showed a low viremia level, whereas plasma and additional CSF collected during the convalescent phase 5 and 2 days later, respectively, were negative (Figure 2, panel B). A second confirmatory assay targeting the RNA-dependent RNA polymerase gene confirmed all positive results (data not shown). Plasma and fecal specimens collected from the patient 8 months later were negative (Figure 2, panel B).

**Figure 3:**
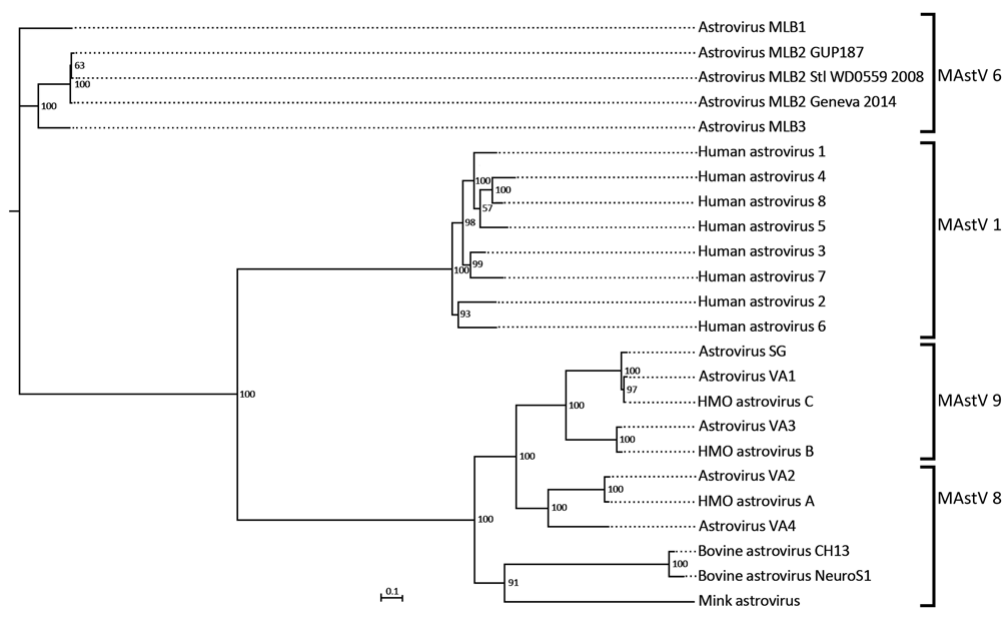
Phylogenetic tree constructed on the basis of full-length sequences of astroviruses and mamastroviruses. The sequence from the case-patient in this study is astrovirus MLB2 Geneva 2014. Brackets indicate the 4 Mamastrovirus species (MAstV 1, 6, 8, 9) from humans. Virus names and corresponding GenBank accession numbers are listed in Technical Appendix 2 Table. Scale bar indicates nucleotide substitutions per site.

Phylogenetic analysis was performed on the full-length genome and on capsid sequences (Figure 3; Technical Appendix 2 Figure, Table 1). Astrovirus MLB2 Geneva 2014 shows 98.5% nucleotide sequence identity homology with the complete genome of an astrovirus MLB2 isolate MLB2/human/Stl/WD0559/2008 detected in a viremic child in St. Louis, Missouri, USA, in 2011 (*11*).

### Pilot Prevalence Study

A total of 943 fecal specimens from 615 unique patients were screened; specimens from 6 patients (1%) were positive for astrovirus MLB2 by the 2 RT-PCR assays, bringing the overall number of positive patients to 7. Except for 1 immunocompetent infant who was brought for treatment with diarrhea of 15 days’ duration, all patients were highly immunocompromised: 1 was an adult recipient of a hematopoietic stem cell transplant (HSCT) (Figure 2, panel C) and 4 were children who received solid organ transplants (Figure 2, panels D–G). Two patients had concomitant viremia (Figure 2, panels C and D), and 1 had 2 astrovirus MLB2–positive fecal samples, collected 2 weeks apart (Figure 2, panel G). Most patients had past or current digestive tract symptoms; the immunocompetent infant with diarrhea had no other digestive pathogen retrieved, and no other explanation was found for his symptoms. One child who had received a transplant experienced concomitant and recurrent *Clostridium difficile* infection, and adenoviral DNA was found in feces.

Among 424 CSF specimens collected from 404 patients hospitalized in the 2 previous years, 1 supplementary specimen was positive for astrovirus MLB2. The patient was the HSCT recipient whose feces had also been screened positive (Figure 2, panel C). Of note, we detected astrovirus MLB2 RNA in CSF from this patient over a 3-month period and intermittently in plasma specimens from this patient over a 2-month period.

### Meningitis: Clinical Case Descriptions

The case-patient (Figure 2, panels A, B) had been enrolled in the virus discovery study. In September 2014, this previously healthy 21-year-old woman sought treatment for an unusually severe headache and fever of a few hours’ duration. She lived in a rural area, had 2 housecats, and had recently traveled to Portugal. She worked at a children’s daycare center. Physical examination revealed neck stiffness without focal neurologic deficits. Blood laboratory test results were within reference limits; leukocyte count was 4.2 × 10^9^ cells/L, and C-reactive protein level was 27 nmol/L. Analysis of CSF obtained by lumbar puncture (LP) at admission revealed clear fluid yet an abnormally high leukocyte count of 915 cells/μL (reference range 0–5 cells/μL), with 93% neutrophils; slightly elevated protein (73 mg/dL, reference range 15–45 mg/dL); and a CSF/plasma glucose ratio of 0.53. The patient was admitted and ceftriaxone and acyclovir were administered empirically. Bacterial CSF cultures remained negative, as did viral real-time RT-PCR assays targeting herpes simplex virus, varicella-zoster virus, enteroviruses, parechovirus, and Toscana virus. Serologic testing for HIV, *Treponema pallidum* (syphilis), tickborne encephalitis virus, and *Borrelia burgdorferi* (Lyme disease) were negative, as were blood cultures. The patient underwent repeat LP 4 days after admission; the CSF leukocyte count had decreased to 47 cells/μL with a shift toward lymphocytic predominance (92%), whereas protein levels had returned to reference range (21 mg/dL). Real-time PCR results for herpes simplex virus and varicella-zoster virus remained negative on the second CSF analysis, and acyclovir was discontinued. Real-time PCR results for *Streptococcus pneumoniae* and *Neisseria meningitidis* was also negative, and ceftriaxone was discontinued after 7 days. The patient continued to improve; she was discharged 10 days after admission with a presumptive diagnosis of viral meningitis.

The second patient (Figure 2, panel C) was a HSCT recipient screened by the pilot prevalence study. He was a 37-year-old man who underwent HSCT on October 2014 for acute myeloid leukemia. His household included young children. In March 2015, he experienced a headache and was ultimately given a diagnosis of a leukemic relapse, including meningeal involvement with a CSF leukocyte count of 2,240 cells/μL and a flow cytometry result confirming that 90% were blast cells. Magnetic resonance imaging showed signs of meningeal leukemic infiltration without cerebral involvement. The patient subsequently received 6 cycles of intrathecal chemotherapy and 4 cycles of 5-azacitidine, which led to remission. LPs on March 25 and June 3 revealed CSF leukocyte counts within normal limits that were nonetheless difficult to interpret given the patient’s marked systemic leukopenia (0.7 and 1.1 × 10^9^ cells/L, respectively). At that time, the patient experienced episodes of vertigo, limb weakness, lightheadedness, and recurrent headache, for which follow-up magnetic resonance imaging was performed. Although meningeal infiltration had diminished, it was still detectable. Thus, the patient underwent cranial irradiation beginning in July 2015. The patient then received a diagnosis of a second relapse of leukemia and finally died in December 2015. 

## Discussion

Detection of astrovirus MLB2 RNA in the CSF of the initial case-patient with acute meningitis highlights the conclusion that as-yet-unrecognized potential new human pathogens can be identified by means of molecular unbiased screening in appropriately targeted populations. The subsequent detection of the same virus’s RNA in fecal specimens of 6 additional patients (of whom 5 were immunocompromised, 2 had viremia, and 1 had a positive CSF specimen) demonstrates that this virus circulates in the community and could be an unrecognized cause of certain clinical manifestations, particularly in patients at increased risk for complications.

Although several studies have demonstrated that novel species of human astroviruses are circulating throughout the world, their associated clinical manifestations require further characterization. To the best of our knowledge, 5 cases of astrovirus CNS infection have been reported in humans, 1 caused by classic HAstV-4 and 4 caused by HAstV-VA1/HMO-C/PS (Table), but none attributed to the distant astrovirus MLB2 described here. In 2011, Wunderli et al. described a cluster of 3 children in a pediatric stem cell transplantation unit who were infected by classic HAstV-4 (*12*). Disseminated viral infection was diagnosed in 1 child who died of multiple organ failure; astrovirus was detected in several organs, including the brain and bone marrow. Similarly, HAstV-VA1/HMO-C has been detected in a few immunocompromised persons who had CNS infection and encephalitis (*13*–*16*). In animals, 2 astroviruses closely related to HAstV-VA1/HMO-C have been identified, 1 in minks with so-called shaking mink syndrome, the other in cattle with nonsuppurative encephalitis (*28*–*30*).

**Table:** Clinical cases of astrovirus infection recovered outside the digestive tract in humans by next-generation sequencing or real-time RT-PCR*

Authors (reference or figure)	Astrovirus strain	Species	Sample analyzed and results					
Brain biopsy/CSF	Plasma/ serum	Feces	Urine	NPS	Other
Holtz et al. (*11*)	HAstV MLB2	MAstV 6	NP	+	NP	NP	+	NP
This study	HAstV MLB2	MAstV 6						
Case-patient (Figure 2, panels A, B)			+	+	+	+	–	NP
HSCT recipient (Figure 2, panel C)			+	+	+	NP	NP	NP
Patient D (Figure 2, panel D)			NP	+	+	NP	NP	NP
Wunderli et al. (*12*)	Classical HAstV serotype 4	MAstV1						
Patient 1			–/NP	+	+	–/NP	+	-/NP
Patient 2			+	+	+	–/NP	–/NP	+†
Patient 3			+	–/NP	–/NP	–/NP	+	–/NP
Quan et al. (*13*)	HAstV-PS	MAstV 9	+	NP	NP	NP	NP	–‡
Brown et al. (*14*)	HAstV-VA1/HMO-C-UK1	MAstV 9	+	+	+	NP	NP	NP
Naccache et al. (*15*)	HAstV-VA1/HMO-C-UK1	MAstV 9	+	NP	NP	NP	NP	NP
Fremond et al. (*16*)	HAstV-VA1/HMO-C-PA	MAstV 9	+	NP	NP	NP	NP	NP

*RT-PCR, reverse transcription PCR; CSF, cerebrospinal fluid; NPS, nasopharyngeal swab; HAstV, human astrovirus; MAstV, mamastrovirus; NP; not performed; +, positive; –, negative; HSCT, human stem cell transplant. †Vesicle swab, heart, lung, spleen, bone marrow, kidney, small intestine.  ‡Kidney, liver, spleen.

We have been able to partially fulfil the criteria proposed by Fredricks and Relman to show microbial disease causation on the basis of molecular tests (*31*): the high viral load observed in the fecal specimens of the 2 patients with meningitis suggests that the gastrointestinal tract is the primary site of replication, which is consistent with the tropism of this family of viruses. From the anal swab specimen of the initial case-patient, we were able to sequence the whole genome and thus demonstrate the presence of the entire virus. The transient presence of viral RNA in plasma and CSF with cycle threshold values indicating a lower viral load suggests hematogenous dissemination from the gastrointestinal tract to the meninges. With resolution of the disease, blood and fecal samples were negative for HAstV MLB2 RNA. Thus, a causal link between astrovirus MLB2 and the case-patient’s acute meningitis is highly plausible. In contrast to patients in previous reports, the case-patient in our study was immunocompetent with an uncomplicated clinical course, suggesting that these viruses should probably not be considered as purely opportunistic.

In the HSCT recipient, the protracted, relatively high viral loads detected in plasma and CSF potentially mirrored the cycles of immunosuppressive therapy he concomitantly received. Yet, other factors, such as his underlying illness or potential drug toxicity, could have caused and maintained his neurologic symptoms. Nonetheless, our observations indicate that astroviruses cause viremia and express CNS tropism; these findings provide a plausible explanation for the encephalitis cases recently described (*12*–*16*). The source of infection in these patients is unknown, although they may have been infected by contact with children. The additional detection of the virus in 5 fecal specimens from children supports this hypothesis. Alternatively, although no animal astrovirus MLB2 reservoir has yet been identified, zoonotic transmission remains another possibility (*17*,*32*).

We assessed the potential circulation of this unrecognized virus in humans by examining its prevalence in different biological specimens of interest. Our hospital-based investigation over a 1-year period found an incidence rate of astrovirus MLB2 infection of 1.1% (including the case-patient) in feces, which is higher than found in most other studies (*3*,*9*,*33*,*34*). In comparison, in our hospital, 1-year incidence rates of 3 other enteric viruses, noroviruses, rotaviruses, and enteroviruses, were 5.5%, 6.7%, and 2.7%, respectively. Whether the global prevalence of astrovirus MLB2 is underestimated or fluctuates from year to year remains to be determined. Unlike results from a previous report (*14*), CSF samples were successfully screened, with a positivity rate of 0.5% (2/405), which supports consideration of the virus in the investigations of unexplained CNS infection. Curiously, a classic symptom of human astrovirus infection is headache (*2*,*4*).

The pathophysiology and clinical manifestations of astrovirus MLB2 and other astroviruses require further definition. Of 7 patients with astrovirus MLB2 in feces, in only 1 patient did this finding have a clear clinical correlation with digestive symptoms. Thus, as with noroviruses (*35*), carriage may be prolonged after a subclinical or transient gastrointestinal illness or, as with classic astroviruses or enteroviruses, gastrointestinal replication and carriage may occur without digestive symptoms. Indeed, in a recent case-cohort study, astrovirus MLB2 was recovered in the feces of 8 patients who did not have overt digestive symptoms (*9*).

Additional virologic and epidemiologic investigations are required to assess our findings; however, seroresponses could not be evaluated because of the lack of an available antibody assay. In the absence of neural tissue sampling, in situ hybridization could not be considered. We could not isolate or demonstrate active viral replication because of the lack of a cell culture system for novel astroviruses. Furthermore, our RT-PCR assays were not quantitative, although cycle threshold values gave substantial information. These factors require more laboratory investigations, which are justified by the potential clinical effects of astroviruses that this study has highlighted. Finally, our prevalence study was retrospective and did not include healthy control patients, limiting our ability to draw solid conclusions with regard to associated disease patterns.

Although we do not provide evidence of disease causality for HAstV MLB2, according to classic Koch’s postulates, our preliminary findings could place astrovirus MLB2 in the differential diagnosis not only of diarrhea but also of aseptic meningitis and protracted infection in highly immunocompromised hosts. Potential determinants of extraintestinal dissemination, such as viral load kinetic, immune response, and host and viral genetic factors, require further characterization. Should further studies confirm our findings, patients with unexplained meningoencephalitis and those with severe immunosuppression should be considered for astrovirus MLB2 screening.

Technical Appendix 1 Protocol for investigation of central nervous system/respiratory diseases of unrecognized viral etiology.

Technical Appendix 2 Two tables showing astrovirus names and GenBank accession numbers used for a phylogenetic tree based on full-length sequences and a phylogenetic tree based on capsid sequences. The Figure shows a phylogenetic tree of astroviruses based on capsid sequences.
