# Supplementary material for: Astrovirus MLB2, a New Gastroenteric Virus Associated with Meningitis and Disseminated Infection
Source: Emerg Infect Dis. 2016 May;22(5):846–53. doi: 10.3201/eid2205.151807 (PMC4861523; doi:10.3201/eid2205.151807)
Supplement: Technical Appendix 1 — Protocol for investigation of central nervous system/respiratory diseases of unrecognized viral etiology. [file 15-1807-Techapp-s1.pdf]

Article DOI: http://dx.doi.org/10.3201/eid2205.151807

Astrovirus MLB2, a New Gastroenteric Virus Associated with Meningitis and Disseminated Infection

Technical Appendix 1

Hôpitaux Universitaires de Genève

Laboratoire de Virologie
Service de médecine de laboratoire
Département de médecine génétique et de laboratoire
Service des maladies infectieuses
Département des spécialités de médecine
Prof. Laurent Kaiser
Secteur microbiologie

Geneva, August 25th 2015

Investigation of central nervous system/respiratory diseases of unrecognized viral etiology

A. Background

Viral nucleic acid-based identification has changed the face of clinical virology during the last decade. Screening for common viral infections by polymerase chain reaction (PCR) or reverse transcription (RT-PCR) is performed on a daily basis and, if needed, viral loads can be quantified. Although a specific virus can be targeted individually, it is also common practice to use panels containing multiple targets adapted to specific syndromes. These panels can be restricted to a handful of viruses in the case of central nervous system (CNS) infections or can target more than 17 different agents in the case of respiratory tract infections. Despite this progress, it is still common for infectious disease specialists to be faced with cases for which a viral disease is suspected, but where all microbiological investigations remain negative or incomplete. Indeed, up to 40% of encephalitis cases, presumably of infectious origin remain, of unknown etiology despite extensive microbial investigations. Although observed in a lower extent, the same situation is noted in studies investigating the cause of pneumonia or lower respiratory tract infections. This problematic is clearly demonstrated in the following table that illustrates the total number of CSF and bronchoalveolar lavages (BAL) received in our routine laboratory in 2011 and the respective positivity rate.

	CSF	BAL
Total number of cases	477	245
% Positivity	**18.2 %**	**23.2 %**

Next-generation sequencing (NGS) technologies are evolving very rapidly and have provided the most powerful tools for discovering previously unrecognized human pathogens. Originally, NGS became available in 2005 and was widely used for whole genome sequencing due to its ability to rapidly generate vast amounts of sequence information. Since then, several NGS platforms based on different biochemistry and sequencing protocols have evolved and are currently commercially available (Illumina, 454 pyrosequencing, Ion Torrent, SOLiD, etc.). Pertinent to our investigation discussed below, NGS has been used for key investigations in clinical virology enabling the discovery of several novel viruses, such as:

1. Two recently identified arenaviruses responsible for patient deaths after solid organ transplantation and hemorrhagic fever.
2. A new polyomavirus (MCPyV) associated with most Merkel cell carcinomas.
3. A new phlebovirus associated with severe febrile illness in the USA.
4. A new enterovirus genotype identified in stool samples from children with acute flaccid paralysis.
5. A novel picornavirus associated with gastroenteritis.
6. New influenza H3N2 variant virus of swine origin.
7. The very recent identification (September 2012) of a novel coronavirus resulting in acute respiratory distress syndrome or patient death in subjects that had travelled in Saudi Arabia.

B. Goal

Our study will use NGS technologies as a tool for the discovery of new virus or distant variants from specimens collected on 4 years (starting in June 2013) in the University Hospital of Geneva from adults/pediatrics patients for whom a meningo-encephalitis is diagnosed and etiology remains unknown. Although the causative agents of acute meningo-encephalitis have already been investigated in different studies, very few have conducted systematic pre-established microbiological guideline. The identification of each case in a timely manner, together with the systematic storage of biological specimens other than plasma and CSF, will increase our ability to investigate appropriately each case. Beyond the technology, our aim is to link NGS to relevant investigations in the field of clinical virology. But most importantly, this can also be done due to our access to very relevant clinical specimens in highly selected cohort of patients. In the case of identification of novel virus sequences, this study will enable to develop novel diagnostic tools, such as specific real-time (RT)-PCR assays. Similarly, we will also include patients presenting a clinical syndrome of probable infectious etiology without identified causative microorganism despite complete investigations (i.e.: pericarditis, pleural effusion, pneumonia, hepatitis, fever of unknow origin).

C. Protocol

Identification of patient, consenting of adult patient or parents/guardians and enrolment :

This investigation will include patient (adults and children) presenting to our institution with a clinical diagnosis of meningitis, meningo-encephalitis or a clinical syndrome suspected to be associated to a pathogen agent. Only patient for which the first line of microbiological investigations remain negative will be included in this study. Physician in charge will describe the purpose and the procedure of this study, possible risk/benefits, the rights and the responsibilities of participants. If the adult patient or parents/guardians agree to participate, they will be asked to sign and informed consent form (IC) except if estimated without the mental competency to understand the IC. No analysis will be performed before the signature of the IC. The study will enroll patients for a minimum of 4 years starting in June 2013. The estimated sample size over this period is 20-30 patients per year.
Before the design of the study presented here, the investigators from the Laboratory of Virology have previously identified and selected cases of meningitis, or meningo-encephalitis, as well as others clinical cases for which an infection is highly suspected but remain of unknown etiology despite extensive microbiological analysis. These specimens will be included in this study.

Study specimen collection :

Enrolment:
At enrolment the following specimen will be collected:

- Lumbar puncture will be performed in case of suspicion of meningitis/meningo-encephalitis and only for medical reasons according to the University of Geneva Hospitals guidelines and practice and will not be performed specifically for the study. When lumbar puncture is performed for normal clinical care, the non-used volume will be stored for the study purposes' if available. For pediatric patients, the maximum volume will be collected according to patient's weight, following the standard of care.

- EDTA plasma and serum collection will be performed only for medical reasons according to the University of Geneva Hospitals guidelines and practice and will not be performed specifically for the study. When EDTA plasma or serum collection is performed for normal clinical care, the non-used volume will be stored for the study purposes' if available. Optional, additional EDTA plasma and serum specimen will be collected in a separate vial for study purposes'.

- If not collected for normal clinical care, a nasopharyngeal swabs, a stool (or rectal swab) and urine specimen will be collected for study purposes'.

- Other samples such as bronchoalveolar lavages, pericardial, pleural, or any other sterile fluid might be eventually analyzed for selected cases. All these samples will be exclusively collected for medical reasons.

- Other samples such as bronchoalveolar lavages, pericardial, pleural, or any other sterile fluid might be eventually analyzed for selected cases. All these samples will be exclusively collected for medical reasons.

Samples will be used exclusively for the detection of new pathogen agents. No human genetic study will be performed.

After obtaining the patients agreement, our study requires planning two supplementary visits per patient. The first one is dedicated to collect specimen specifically for this project by the study medical doctor or nurse in case of absence of pathogen agent identification after initial screening performed by routine assays and will be done within the 24 hours after obtaining routine assays results. The second visit is optional and is planned 30 days after discharge to follow-up clinical evolution.

While hospitalized or Follow-up:
Any specimen collected for medical reasons during the hospitalization will be stored after analysis and could be used for the purpose of this study if needed. During hospitalization, blood (optional), urine, respiratory swab and stool specimen may be performed specifically for the purpose of this study after agreement of the patient.
Optional, an additional convalescent plasma specimen (EDTA tube) will be collected on the day of discharge and day 30. Patient will be asked to come back to the hospital if discharged.

Time frame:

The study will enroll patient immediately after ethical approval and will be conducts for a minimum of 4 years.

Study organization:

The study will be coordinated by investigators from the Laboratory of Virology, pediatricians in charge of the emergency division and infectious diseases unit and neurologists from the University of Geneva Hospitals. Investigator will be in charge to organize in their relative units the specimen collection mentioned above.

Laboratory of Virology	Prof Laurent Kaiser (main investigator), Dr Samuel Cordey, Dr Manuel Schibler, Lara Turin and Dr Diem-Lan Vu Cantero
Infectious diseases Service	Prof Laurent Kaiser
Pediatric Department	Prof Klara Posfay-Barbe
Pediatric emergency Division	Prof Alain Gervaix
Pediatric neurology	Dr Joël Fluss and Dr Christian Korff
Adult neurology	Prof Patrice Lalive
Adult emergency room	Olivier Rutschmann
General Medicine	To be determined

Case report form (CRF):

Patient information relevant to the viral infection of unknown etiology will be recorded on an individual CRFs (see enclosed document) designed for this study. Data will be entered on the day of enrolment and during follow-up by investigators. The information contained within the CRFs will be transferred to a computerized database and will be available exclusively to the study team.

Biological Specimen:

Original specimen collected for the purpose of this study will be stored at the appropriate temperature (70°C for CSF, plasma, nasopharyngeal swab, stool, urine specimen and other samples; -20°C for serums) within 24 hours and aliquoted as indicated in specific SOPs. Samples collected will be used for the purpose of this study as stated in the protocol and stored for future use. After specimen analysis, the non-used volumes will be stored by the investigators throughout the period dedicated to the study. Any proposed plans to use samples other than for those investigations detailed in this protocol will be submitted to the relevant ethics committees prior to any testing.

Fortuitous discovery of others viral infection:

In case of fortuitous discovery of viral infection(s), the result will be communicated to Prof. Laurent Kaiser, Dr Klara Posfay-Barbe, Prof. Patrice Lalive or Dr Joël Fluss, and an additional physician from the Infectious diseases Service from the University of Geneva Hospitals not involved in this study. This physician will evaluate the clinical relevance of the fortuitous discovery and decide to transmit or not the information to the patient's doctor. This latter will be in charge to contact directly his patient.

Additionally, in case of discovery of a pertinent and/or novel viral infection, further retrospective investigations on stored biological specimens of other patients will be performed in order to understand the virus' prevalence, pathogenesis and associated clinical syndrome. In case of positive results, clinical data of concerned patients will be reviewed by the infectious diseases specialist and potentially the medical doctor in charge. In case of publication, all data will be anonymously provided.

Specific SOP:

ENROLMENT

Potential participants are patients admitted to Emergency Department , the Intensive Care Unit (ICU), Infectious Diseases Ward or in the General Medicine, Neurology, Cardiology, Pneumology or Gastroenterology.<br><br>The medical doctor in charge and the investigators will evaluate the following screening criteria:<br><br>INCLUSION:<br><br>1. Case with clinical syndrome of probable infectious etiology<br>2. Absence of pathogen agent identification after initial screening performed by routine assays.<br>3. Informed consent given by adult patients, parent or legal guardian<br><br>The medical doctor in charge and the investigators (MD and study nurse) will discuss the study with the adult patient, parents or legal guardians of potential participants and request informed consent. Parents or legal guardians who agree will sign the IC. Once the IC signed, a dedicated CRF will be completed.	
**Anonymous CRF**<br><br>The medical doctor in charge and the investigators will interview and examine the patient and will fill the CRF.<br>The medical doctor in charge or nurse will collect and store the biological samples as described below and record this on the CRF.	
**Specific inclusion criteria for meningitis or meningo-encephalitis:**<br>1. Case with clinical diagnosis of meningitis or meningo-encephalitis<br>2. No contra-indication to perform a lumbar puncture for CSF collection<br>3. Absence of pathogen agent identification in CSF after initial screening performed by routine assays.<br>4. Informed consent given by adult patients, parent or legal guardian<br><br>**Initial screening routine assays available in the laboratory for meningitis and meningo-encephalitis**<br><br>Serological tests to screen for the following acute primary infections:<br>o EBV, CMV, HHV6, HIV, mumps, TBE, *Borrelia*.<br>PCR and RT-PCR assays:<br>o CSF: HSV1 and 2, VZV, enterovirus, parechovirus and HHV6.<br>o Respiratory specimens: for all known respiratory viruses (influenza, RSV, parainfluenza, coronavirus, metapneumovirus, rhinovirus, enterovirus, adenovirus, bocavirus).<br>o Stools: rotavirus, norovirus, astrovirus, sapovirus, enterovirus, parechovirus, and adenovirus.	

The screening assays will not be taken in charge by the study fundings.<br><br>**Further screening potentially performed for the study purposes**<br><br>According to epidemiological factors, the following analysis will be systematically conducted:<br>o Serology in blood: West Nile, Japanese encephalitis, Toscana.<br>o RT-PCR: LCMV and Toscana virus in CSF.<br>According to initial results, serological screening using CSF itself will be completed whenever needed according to recognized procedures (Lyme, VZV, HSV mainly).<br>These additional screening will be taken in charge by the study fundings' (Annex 1).	
**RESEARCH SWABS**<br><br>The medical doctor in charge or nurse will collect<br><br>1. A nasopharyngeal swab in viral transport medium (if none previously collected for normal clinical care).<br>2. A stool specimen (or rectal swab) in a stool container (if none previously collected for normal clinical care).<br>3. A urine specimen in appropriate vial (specifically collected for the study purposes').<br><br>The study medical doctor or nurse will complete the fields of the appropriate labels and label the samples, and will complete one row on the sample log form on the ward.	**Note:**<br><br>Sample swabs, VTM, stool containers, urine vial supplied by HUG
**LUMBAR PUNCTURE**<br><br>The medical doctor in charge and the investigators will collect cerebrospinal fluid (CSF) in case of meningitis or meningoencephalitis as needed for normal clinical care. If available, the non-used volume will be stored for the study purposes'.<br><br>The study medical doctor or nurse will complete the fields of the appropriate labels and label the samples, and will complete one row on the sample log form on the ward.	**Note:**<br><br>Lumbar puncture kits and tubes supplied by HUG
**BLOOD SAMPLES**<br><br>The medical doctor in charge or nurse will collect blood samples (plasma, serum) as needed for normal clinical care. If available, the non-used volume will be stored for the study purposes'. If the plasma or serum non-used volume is < 1ml, a minimum of 1ml blood sample in an EDTA tube and a minimum of 1ml blood sample in a serum tube will be collected for the study purposes'.<br><br>The study doctor or nurse will complete the fields of the Day 0 EDTA/Serum label and label the samples, and will complete one row on the sample log form on the ward.	**Note:**<br><br>EDTA tubes, serum tube supplied by HUG

**OTHER SAMPLES**<br><br>Depending on the clinical presentation, the medical doctor in charge or nurse may collect other samples as needed for normal clinical care. If available, the non-used volume will be stored for the study purposes'.<br><br>Potential other samples are:<br>• Pericardial fluid<br>• Pleural fluid<br>• BAL (bronchoalveolar lavage)<br>• Solid material (i.e.: hepatic biopsy)<br><br>These samples will be collected only if needed for medical indications. Specimens will not be collected only for study purposes. No additional volume will be collected for study purposes.<br><br>The study medical doctor or nurse will complete the fields of the appropriate labels and label the samples, and will complete one row on the sample log form on the ward.	**Note:**<br><br>Storage material supplied by HUG
**STORAGE**<br><br>The nasopharyngeal swab, stool sample (or rectal swab), urine sample, CSF, blood and other specimens will be immediately stored at 4°C in the in the fridge. The doctor in charge has the responsibility to ensure that all collected samples and the sample log forms will be transferred to the Laboratory of Virology at the end of each working day (5 pm) and on Monday mornings (9 am) for storage at -70 °C.<br>As soon as the Laboratory of Virology receive the samples and the sample log forms, investigators have the responsibility to aliquot (CSF = 250 ul, EDTA tube/Serum = 500ul, Nasopharyngeal swab in VTM = 1 ml, Stool = 1 ml, Urine = 1 ml) and store the samples at -70 °C or -20°C.<br>Specimen will be kept and use only for the presented study. After specimen analysis, any leftover will be stored by the investigators. These specimens will only be used for microbiological investigations as described in our protocol or complementary virological characterization if needed. No human genetic analysis will be done.	
**DISCHARGE**	
**RESEARCH BLOOD**<br><br>Optional, the doctor in charge or nurse will take a blood sample in an EDTA tube for the study.<br><br>The doctor in charge or nurse will complete the fields of the EDTA Discharge label and label the samples, and will complete one row on the sample log form on the ward.	**Note:**<br>EDTA tubes supplied by HUG<br><br>Labels and sample logs supplied by HUG
**FOLLOW-UP**	
Optional, after discharge, the patients will be called by the doctor in charge or by investigators to follow-up clinical evolution. Follow-up visits will be scheduled at day 30 or thereafter if clinically indicated.	

BLOOD SAMPLES	
Optional, the doctor in charge or nurse will take a blood sample in an EDTA tube for the study.<br><br>The doctor in charge or nurse will complete the fields of the EDTA Follow-up label and label the samples, and will complete one row on the sample log form on the ward.	**Note:**<br>EDTA tubes supplied by HUG<br><br>Labels and sample logs supplied by HUG

Additional investigations

Retrospective

Optional, extensive retrospective analysis will be performed on biological specimens retrieved for routine clinical care and stored in any laboratory of the University of Geneva Hospitals or Geneva University of medical school.

Main biological specimens include:

- Stools
- Blood
- CSF fluid
- Biopsies
- Respiratory specimens (NPS, BAL)
- Urine
- Other specimens can be analysed depending on the virus' tropism.

Analysis performed:

- Molecular diagnostic tests (PCR, NGS)
- Antigen or antibody detection
- Culture

In case of a positive result, clinical data will be reviewed by the infectious diseases specialist in charge of the investigation.

Prospective

In case of prospective additional investigation concerning other patients than the case patient issued from the initial study protocol, a separate protocol or novel amendment will be submitted to the local ethics committee.

References

1. Capobianchi, M.R., Giombini, E., Rozera, G., 2013. Next-generation sequencing technology in clinical virology. Clin. Microbiol. Infect. 19**,** 15-22.

2. Cordey, S., Junier, T., Gerlach, D., Gobbini, F., Farinelli, L., Zdobnov, E.M., Winther, B., Tapparel, C., Kaiser, L., 2010. Rhinovirus genome evolution during experimental human infection. PLoS. ONE. 5**,** e10588.

3. de, O.F., Avellon, A., Echevarria, J.E., Sanchez-Seco, M.P., Trallero, G., Cabrerizo, M., Casas, I., Pozo, F., Fedele, G., Vicente, D., Pena, M.J., Moreno, A., Niubo, J., Rabella, N., Rubio, G., Perez-Ruiz, M., Rodriguez-Iglesias, M., Gimeno, C., Eiros, J.M., Melon, S., Blasco, M., Lopez-Miragaya, I., Varela, E., Martinez-Sapina, A., Rodriguez, G., Marcos, M.A., Gegundez, M.I., Cilla, G., Gabilondo, I., Navarro, J.M., Torres, J., Aznar, C., Castellanos, A., Guisasola, M.E., Negredo, A.I., Tenorio, A., Vazquez-Moron, S., 2012. Viral infections of the central nervous system in Spain: A prospective study. J. Med. Virol.

4. Granerod, J., Ambrose, H.E., Davies, N.W., Clewley, J.P., Walsh, A.L., Morgan, D., Cunningham, R., Zuckerman, M., Mutton, K.J., Solomon, T., Ward, K.N., Lunn, M.P., Irani, S.R., Vincent, A., Brown, D.W., Crowcroft, N.S., 2010. Causes of encephalitis and differences in their clinical presentations in England: a multicentre, population-based prospective study. Lancet Infect. Dis. 10**,** 835-844.

5. Grard, G., Fair, J.N., Lee, D., Slikas, E., Steffen, I., Muyembe, J.J., Sittler, T., Veeraraghavan, N., Ruby, J.G., Wang, C., Makuwa, M., Mulembakani, P., Tesh, R.B., Mazet, J., Rimoin, A.W., Taylor, T., Schneider, B.S., Simmons, G., Delwart, E., Wolfe, N.D., Chiu, C.Y., Leroy, E.M., 2012. A novel rhabdovirus associated with acute hemorrhagic fever in central Africa. PLoS. Pathog. 8**,** e1002924.

6. Lysholm, F., Wetterbom, A., Lindau, C., Darban, H., Bjerkner, A., Fahlander, K., Lindberg, A.M., Persson, B., Allander, T., Andersson, B., 2012. Characterization of the viral microbiome in patients with severe lower respiratory tract infections, using metagenomic sequencing. PLoS. ONE. 7**,** e30875.

7. Mailles, A., Stahl, J.P., 2009. Infectious encephalitis in france in 2007: a national prospective study. Clin. Infect. Dis. 49**,** 1838-1847.

8. McMullan, L.K., Folk, S.M., Kelly, A.J., MacNeil, A., Goldsmith, C.S., Metcalfe, M.G., Batten, B.C., Albarino, C.G., Zaki, S.R., Rollin, P.E., Nicholson, W.L., Nichol, S.T., 2012. A new phlebovirus associated with severe febrile illness in Missouri. N. Engl. J. Med. 367**,** 834-841.

9. Zaki, A.M., van, B.S., Bestebroer, T.M., Osterhaus, A.D., Fouchier, R.A., 2012. Isolation of a novel coronavirus from a man with pneumonia in Saudi Arabia. N. Engl. J. Med. 367**,** 1814-1820.

Laboratoire de Virologie

Service de médecine de laboratoire
Département de médecine génétique et de laboratoire

Service des maladies infectieuses
Département des spécialités de médecine
Prof. Laurent Kaiser
Secteur microbiologie

Genève, le 25 août 2015

FORMULAIRE D'INFORMATION ET DE CONSENTEMENT (version 4)

Madame, Monsieur,

Vous êtes invité à participer à une étude intitulée « ***Recherche des nouveaux agents viraux associés aux méningites, méningo-encéphalites et autres infections d'origine indéterminée***» car vous, votre enfant ou votre proche présentez une infection pour laquelle aucun microbe n'a été identifié.

Le présent document vous renseigne sur les modalités de ce projet de recherche. Pour participer à ce projet, vous devrez signer le consentement à la fin de ce document et nous vous en remettrons une copie signée et datée.

Informations générales sur l'étude clinique et son financement

Cette étude a pour objectif de mettre en évidence d'éventuels nouveaux virus associés aux infections virales. A ce jour, pour un grand nombre d'infections, il est impossible pour les laboratoires d'analyses d'identifier le microbe responsable (p.ex. virus, bactérie, champignon). Ceci s'explique par le nombre important de microbes pouvant être en cause et aussi par le fait que probablement un certain nombre de microbes restent à découvrir. Cette recherche sera réalisée via les échantillons de sang et ainsi que d'autres prélèvements effectués pour effectuer le diagnostic initial de l'infection selon les règles habituelles. Des prélèvements supplémentaires, tels que des frottis de gorge, selles ou urines pourront être spécifiquement effectués pour cette étude. Cette étude est menée au sein des différents services des HUG assistant des patients adultes ou pédiatriques.

Cette étude est financée par le Fonds National Suisse de la recherche scientifique.

Objectifs du projet

Cette étude a pour principal objectif de rechercher et identifier des nouveaux virus responsables d'infections dont l'origine reste indéterminée malgré des investigations complètes.

Déroulement de l'étude

Vous serez traité et suivi conformément à la prise en charge standard en vigueur dans notre établissement pour ces infections. Une fois votre accord donné, nous effectuerons des analyses supplémentaires sur l'ensemble des prélèvements collectés initialement pour effectuer le diagnostique de l'infection ainsi que sur les différents frottis effectués spécifiquement pour cette étude. Aucune analyse génétique sur l'ADN humain ne sera réalisée.

En cas de découverte fortuite d'une autre infection virale, les résultats seront analysés par un groupe de spécialistes en infectiologie des Hôpitaux Universitaires de Genève. Ces derniers décideront de la pertinence de cette découverte et transmettrons les résultats considérés utiles à votre médecin traitant qui se chargera si besoin de vous contacter directement.

Droit de retrait sans préjudice de la participation

Il est entendu que votre participation à ce projet de recherche est tout à fait volontaire et que vous restez libre, à tout moment, de mettre fin à votre participation sans avoir à motiver votre décision ni à subir de préjudice de quelque nature que ce soit.

Risques et désagréments

La participation à cette étude ne comporte aucun risque personnel.

Bénéfices

Votre participation pourrait permettre la détection de nouveaux microbes ou virus confirmant le diagnostic de votre infection. Ceci permettra également de développer ou d'inclure de nouveaux tests diagnostics pour la prise en charge future de patients avec une infection d'origine indéterminée.

Confidentialité

Le laboratoire de Virologie et les autres services des HUG impliqués dans l'étude garantissent la confidentialité de toutes les données en conformité avec la législation suisse sur la protection des données.

Les données du projet de recherche pourront être publiées dans des revues scientifiques. Aucune publication ou communication scientifique ne renfermera d'information permettant de vous identifier.

Indemnité

Vous ne percevrez aucune indemnité financière pour participer à cette étude.

Personnes de contact et investigateur principal du projet

En cas de questions pendant ou après l'étude, vous pouvez à tout moment contacter les responsables suivants pour l'étude: Dr. Samuel Cordey 079 553 3645 ou Dr. Manuel Schibler 079 553 4816. Pour les cas pédiatriques, merci de contacter le Prof Klara Posfay-Barbe 079 553 2586.

Pour toute question information complémentaire, l'investigateur principal de cette étude peut être contacté : Prof. Laurent Kaiser 022 372 49 92

Aspect éthique

Le protocole a été approuvé par le Chef de Service et le Comité d'Ethique de la recherche des Hôpitaux Universitaires de Genève.

Consentement du participant

Je, ____ *(nom, prénom)*, déclare avoir lu et/ou compris le présent formulaire et j'en ai reçu un exemplaire. Je comprends la nature et le motif de ma participation au projet. J'ai eu l'occasion de poser des questions auxquelles on a répondu, à ma satisfaction.
Par la présente, je consens librement de participer au projet et affirme avoir eu suffisamment de temps de réflexion pour prendre ma décision.

Date : ________________ Signature du participant : _____________________________

Si requis, consentement de la part du responsable légal ou d'un proche

Je, ____ *(nom, prénom)*, déclare avoir lu et/ou compris le présent formulaire et j'en ai reçu un exemplaire. Je comprends la nature et le motif de participation de mon enfant ou mon proche au projet. J'ai eu l'occasion de poser des questions auxquelles on a répondu, à ma satisfaction.
Par la présente, je consens librement de faire participer mon enfant ou mon proche, ____ *(nom, prénom)* de participer au projet et affirme avoir eu suffisamment de temps de réflexion pour prendre ma décision.

Lien avec le patient : ________________

Date : ________________ Signature: _____________________________

Déclaration du responsable de l'obtention du consentement

Je, ____ *(nom, prénom),* certifie avoir expliqué à la participante ou au participant intéressé(e) les termes du présent formulaire, avoir répondu aux questions qu'il ou qu'elle m'a posées à cet égard et lui avoir clairement indiqué qu'il

ou qu'elle reste, à tout moment, libre de mettre un terme à sa participation au projet de recherche décrit ci-dessus. Je m'engage à garantir le respect des objectifs de l'étude et à respecter la confidentialité.

Date : _______________ Signature : _____________________________

Déclaration de l'investigateur principale de l'étude

Je, Prof. Laurent Kaiser, investigateur principal de l'étude, déclare que les investigateurs de cette étude sommes responsables du déroulement du présent projet de recherche. Nous nous engageons à respecter les obligations énoncées dans ce document.

Signature du chercheur principal de l'étude : _________________________________
