# Supplementary material for: Astrovirus MLB2, a New Gastroenteric Virus Associated with Meningitis and Disseminated Infection
Source: Emerg Infect Dis. 2016 May;22(5):846–53. doi: 10.3201/eid2205.151807 (PMC4861523; doi:10.3201/eid2205.151807)
Supplement: Technical Appendix 2 — Two tables showing astrovirus names and GenBank accession numbers used for a phylogenetic tree based on full-length sequences and a phylogenetic tree based on capsid sequences. The Figure shows a phylogenetic tree of astroviruses based on capsid sequences. [file 15-1807-Techapp-s2.pdf]

DOI: http://dx.doi.org/10.3201/eid2205.151807

Astrovirus MLB2, a New Gastroenteric Virus Associated with Meningitis and Disseminated Infection

Technical Appendix

Technical Appendix 2 Table 1. Astrovirus names and GenBank accession numbers used for full-length sequences phylogenetic tree

Virus name	Accession no.	GI
Astrovirus MLB1	JQ086552.1	GI:380467967
Astrovirus MLB2 Stl WD0559 2008	JF742759.1	GI:354805828
Astrovirus MLB2 GUP187	AB829252.1	GI:523453979
Astrovirus MLB3	NC_019028.1	GI:410428374
Astrovirus SG	GQ891990.1	GI:296932862
Astrovirus VA	NC_013060.1	GI:255357299
Astrovirus VA2	NC_018669.1	GI:407868423
Astrovirus VA3	NC_019026.1	GI:410428372
Astrovirus VA4	NC_019027.1	GI:410428373
Bovine astrovirus CH13	NC_024498.1	GI:667714408
Bovine astrovirus NeuroS1	KF233994.1	GI:514389190
HMO astrovirus A	NC_013443.1	GI:262166845
HMO astrovirus B	GQ415661.1	GI:261597212
HMO astrovirus C	GQ415662.1	GI:261597215
Human astrovirus 1	KF211475.1	GI:548797282
Human astrovirus 2	KF039911.1	GI:542717312
Human astrovirus 3	GU732187.1	GI:291508554
Human astrovirus 4	KF039913.1	GI:542717348
Human astrovirus 5	JQ403108.1	GI:380846546
Human astrovirus 6	GQ495608.1	GI:259121926
Human astrovirus 7	AF248738.2	GI:14572176
Human astrovirus 8	AF260508.1	GI:9230736
Mink astrovirus	NC_004579.1	GI:28867239

Technical Appendix Table 2. Astrovirus names and GenBank accession numbers used for capsid sequences phylogenetic tree

Virus name	Accession no.	GI
Astrovirus MLB1	AFD61563.1	GI:380467970
Astrovirus MLB2 Stl WD0559 2008	AER41414.1	GI:354805831
Astrovirus MLB2 GUP187	BAN62843.1	GI:523453982
Astrovirus MLB3	YP_006905854.1	GI:410493725
Astrovirus SG	ADH93577.1	GI:296932865
Astrovirus VA	YP_003090287.1	GI:255357301
Astrovirus VA2	YP_006792628.1	GI:407868426
Astrovirus VA3	YP_006905860.1	GI:410493719
Astrovirus VA4	YP_006905857.1	GI:410493722
Bovine astrovirus CH13	YP_009047248.1	GI:667714411
Bovine astrovirus NeuroS1	AGO50636.1	GI:514389192
HMO astrovirus A	YP_003275953.1	GI:262166848
HMO astrovirus B	ACX85474.1	GI:261597214
HMO astrovirus C	ACX85476.1	GI:261597217
Human astrovirus 1	AGX15185.1	GI:548797285
Human astrovirus 2	AGV40897.1	GI:542717313
Human astrovirus 3	ADE09295.1	GI:291508557
Human astrovirus 4	AGV40905.1	GI:542717351
Human astrovirus 5	AFE84778.1	GI:380846548
Human astrovirus 6	ACV92107.1	GI:259121929
Human astrovirus 7	AAK31913.1	GI:13603731
Human astrovirus 8	AAF85964.1	GI:9230739
Mink astrovirus	NP_795336.1	GI:28867242

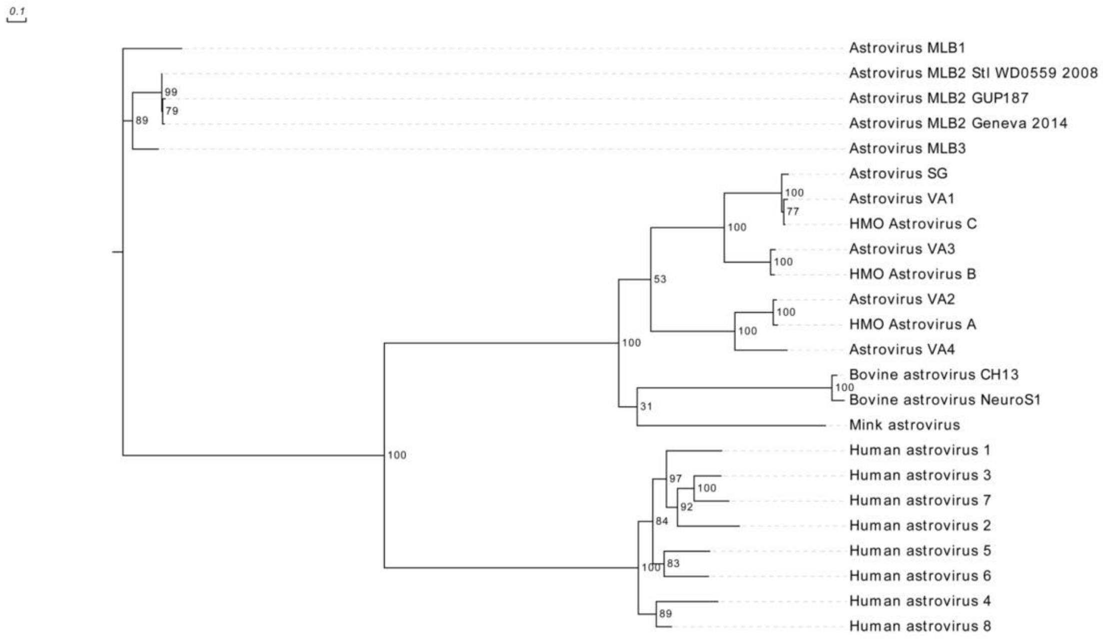

Technical Appendix 2 Figure. Phylogenetic tree of astroviruses based on capsid sequences. (Technical Appendix 2 Table 2 shows correspondence between virus name and accession numbers.)
